# Gut Metabolism Links Precision Nutrition, Exercise, and Healthspan in *Drosophila melanogaster*

**DOI:** 10.1111/acel.70628

**Published:** 2026-07-20

**Authors:** Fangchao Wei, Shiyu Liu, Yudong Sun, Haipeng Huang, Juan Liu, Fengzhong Wang, Bei Fan

**Affiliations:** ^1^ Institute of Food Science and Technology Chinese Academy of Agricultural Sciences Beijing China; ^2^ Department of Pharmacology and Cancer Biology Duke University School of Medicine Durham North Carolina USA; ^3^ Capital Medical University Beijing China; ^4^ Chinese Institutes for Medical Research (CIMR) Beijing China; ^5^ Department of Biochemistry Duke University School of Medicine Durham North Carolina USA; ^6^ Beijing Forestry University Beijing China; ^7^ Institute of Food and Nutrition Development, Ministry of Agriculture and Rural Affairs Beijing China

## Abstract

Longevity‐promoting interventions commonly entail functional trade‐offs, raising the unresolved question of whether lifespan extension necessarily compromises physiological performance. Here, utilizing a chemically defined diet (CDD) in *Drosophila melanogaster* , we systematically evaluated a multimodal intervention combining methionine restriction (MR), taurine supplementation (Tau), and moderate exercise. This combinatorial approach additively extended lifespan, preserved reproductive capacity, and improved locomotor function. Targeted metabolomics with stable isotope tracing revealed increased mitochondrial TCA cycle flux and enhanced gut redox homeostasis, suggesting these as central metabolic features. Notably, *Lactiplantibacillus plantarum* was found to be associated with the observed benefits, and recolonization experiments suggested that it partially accounts for the benefits conferred by MR‐Tau combined with exercise, supporting a causal but partial role. Together, these findings support a “nutrition‐behavior‐microbiota” framework that uncouples the traditional trade‐off between lifespan and functional health, offering new perspectives for promoting healthy aging.

## Graphical abstract

This study reveals that precision nutrition, exercise, and gut microbiota pathways synergistically regulate host metabolism, redox balance, and arginine‐spermidine metabolism, enhancing antioxidant defenses and mitochondrial stabilization to combat aging.

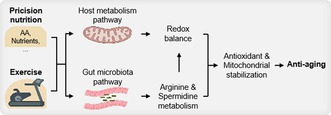

## 1 Introduction

A critical question in aging research is whether lifespan‐extending interventions can preserve essential physiological functions, including physical performance and fertility. Methionine restriction (MR), a dietary intervention that extends lifespan across diverse species, often impairs reproductive capacity and functional fitness, underscoring the need to dissociate beneficial adaptations from adverse trade‐offs (Grandison et al. 2009; Kosakamoto et al. 2023; Lee et al. 2014; Wei et al. 2024). Mitochondria are central mediators of dietary restriction, particularly through the regulation of redox balance and energy metabolism (Gao et al. 2019; Wei et al. 2024; Yaku et al. 2018; Zhang et al. 2016). The gut acts as a critical integrator of nutritional signals, regulating systemic energy balance (Kennedy et al. 2025; Liang et al. 2017; Zhang et al. 2025). Accumulating evidence also implicates the gut microbiota as a potent modulator of host aging and metabolism, broadly impacting physiological functions (Clark et al. 2015; Guo et al. 2015; Ryu et al. 2008; Shin et al. 2011; Storelli et al. 2011). However, how host‐microbiota interactions influence mitochondrial function under precision dietary interventions—and how such crosstalk shapes metabolic trajectories during aging—remains poorly understood.

Taurine supplementation (Tau) and physical exercise have emerged as promising strategies to slow aging. Taurine protects mitochondria and reduces inflammation (Ames 2018; Singh et al. 2023; Suh et al. 2017), whereas exercise enhances metabolic fitness and preserves organ function during aging (Geng et al. 2025; Janssens et al. 2024; Liu, Hua, and Xin 2024; MoTrPAC, S. G 2025). Given their complementary metabolic roles, MR, Tau, and exercise may cooperatively enhance physiological resilience: MR modulates one‐carbon metabolism and redox balance; taurine reinforces mitochondrial and antioxidant systems; and exercise promotes metabolic adaptability. Together, these interventions may mitigate MR‐associated trade‐offs while amplifying its longevity benefits.

However, the integrated metabolic interactions among MR, Tau, and exercise—and the extent to which gut microbiota mediate their combined effects—have not been systematically examined. Here, using *Drosophila melanogaster* maintained on a chemically defined diet (CDD) (Gao et al. 2019; Gu et al. 2022; Wei et al. 2024), we tested the hypothesis that MR‐Tau‐exercise remodels gut mitochondrial redox metabolism through host‐microbiota interactions. By integrating precision nutrition, microbial ecology, targeted metabolomics, and metabolic flux analysis, we delineate how coordinated modulation of diet and behavior reshapes metabolic networks and physiological aging.

## 2 Results

### 2.1 Additive Enhancement of Healthspan by MR and Tau in *D. melanogaster*

**FIGURE 1:**
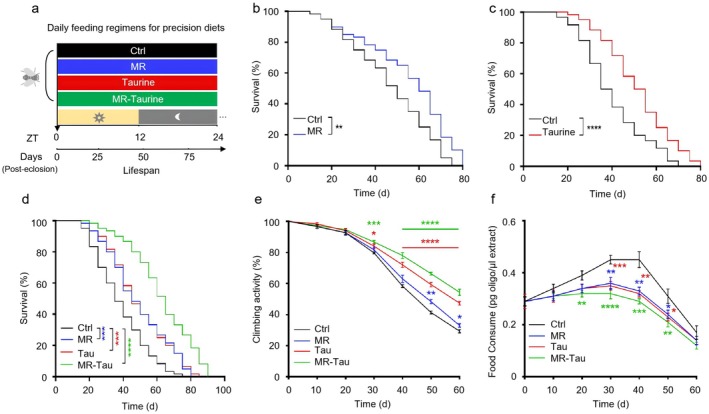
Precision methionine restriction and taurine supplementation enhance healthspan in *Drosophila melanogaster* (a) Schematic representation of the chemically defined precision diets. (b) Survival curves under methionine restriction (MR; 10% methionine). (c) Survival curves with taurine supplementation (Tau; 10 mM). (d) Survival curves under combined dietary conditions (MR + Tau). (e) Climbing performance assay. (f) Food consumption measurements. Survival assays were conducted in parallel male and female virgin cohorts (*n* = 60 per sex per group). Climbing and feeding assays were performed with *n* = 50 flies per group. Survival curves were analyzed using the log‐rank (Mantel–Cox) test. Non‐survival comparisons were evaluated using unpaired two‐tailed *t*‐tests unless otherwise specified. All experiments were independently repeated three times. Data are presented as mean ± s.e.m. Color‐coded asterisks indicate statistical significance relative to control for MR (blue), Tau (red), and MR + Tau (green) groups: *p < 0.05, **p < 0.01, ***p < 0.001, ****p < 0.0001.

**FIGURE A1:**
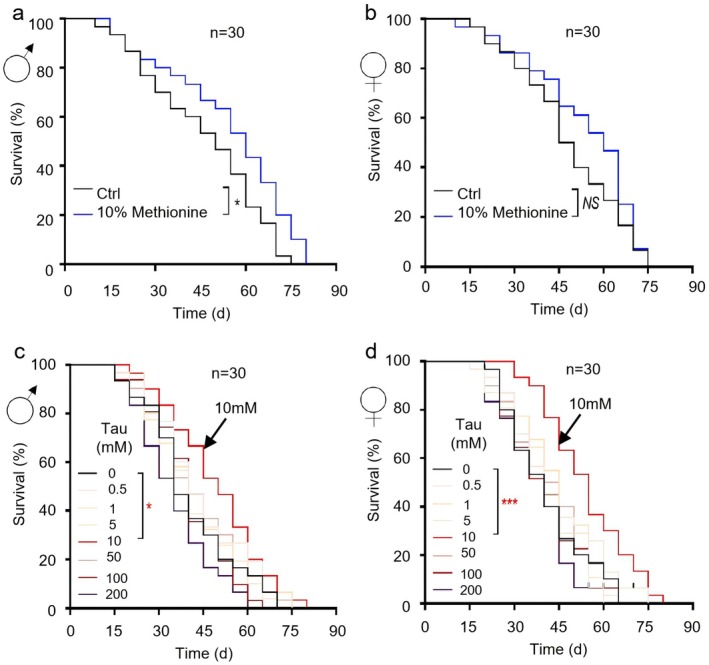
Methionine restriction (MR) and taurine supplementation (Tau) enhance healthspan in *Drosophila*. (a, b) Lifespan of male (a) and female (b) flies under 10% methionine diet. (c, d) Lifespan of male (c) and female (d) flies under taurine supplementation (Tau). Lifespan assays were conducted using parallel cohorts of virgin males and females (*n* = 60 per sex per group). Survival curves were analyzed with the log‐rank (Mantel‐Cox) test. Experiments were independently repeated three times. *p* values are indicated as: NS = *p* > 0.05, *p* < 0.05, **p < 0.01.

To assess the effects of MR, Tau, and their combination (MR‐Tau) on lifespan and functional aging, we designed a feeding scheme based on a CDD system in *Drosophila melanogaster* fruit fly (Figure 1a). Flies were randomized into four dietary groups: control, MR, Tau, or MR‐Tau. As previously established (Wei et al. 2024), MR contained 10% (0.402 mM) of the methionine level in the control diet and significantly extended lifespan (Figure A1a,b, and Figure 1b). Tau showed a dose‐dependent pro‐longevity effect, with 10 mM providing maximal benefit (Figure A1c,d, and Figure 1c). Both MR and Tau increased lifespan in males and females (Figure A1 and Figure 1b,c), and delayed age‐associated locomotor decline (Figure 1e), consistent with previous reports (Du et al. 2021; Smith et al. 2011; Wei et al. 2024).

Methionine is metabolized through homocysteine to cysteine, a precursor for endogenous taurine biosynthesis, with these intermediates playing central roles in redox homeostasis and metabolic regulation (Ripps and Shen 2012). Notably, the MR‐Tau intervention produced additive effects, further extending lifespan (Figure 1d) and improving physical endurance (Figure 1e) beyond either intervention alone.

**FIGURE A2:**
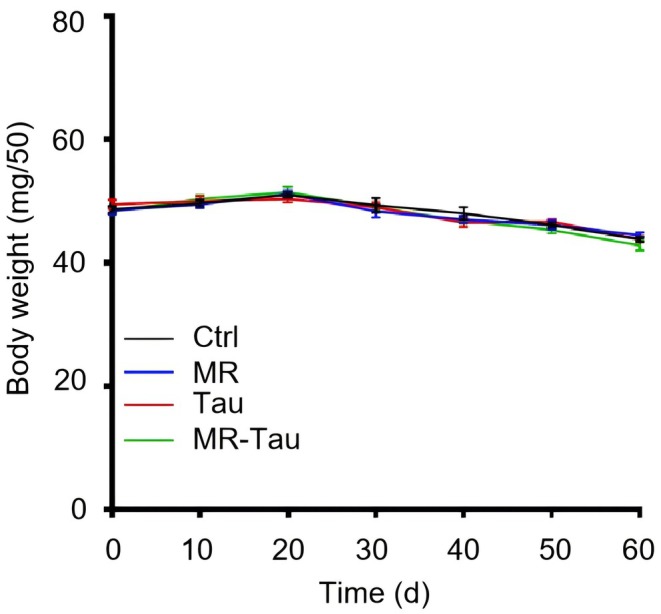
MR and Tau diets do not affect body weight gain. Body weight was measured in adult male and female flies maintained on control, MR, Tau, or combined MR + Tau diets. Measurements were taken at regular intervals throughout the intervention period. A total of 150 flies were analyzed (males, *n* = 100; females, *n* = 50). Each biological replicate consisted of individually measured flies. All experiments were independently repeated three times. Data are presented as mean ± s.e.m.

Considering the brain's role in regulating feeding behavior (Ribeiro and Dickson 2010), and prior evidence that both MR (Bjordal et al. 2014) and taurine (Imae et al. 2014; Kasaoka et al. 2006) modulate hypothalamic activity to alter food intake, we evaluated feeding behavior. Indeed, flies on MR or Tau diets consumed less food, with the greatest reduction observed in the MR‐Tau group (Figure 1f). Despite reduced food intake, body weight remained comparable among groups (Figure A2).

Together, these results suggest that MR and Tau individually promote healthspan by attenuating age‐related declines in survival and locomotor function. Their combination confers additive benefits, supporting a multimodal dietary strategy that enhances healthy aging without compromising physiological integrity.

### 2.2 Additive Dietary Reprogramming of Systemic Metabolism

**FIGURE 2:**
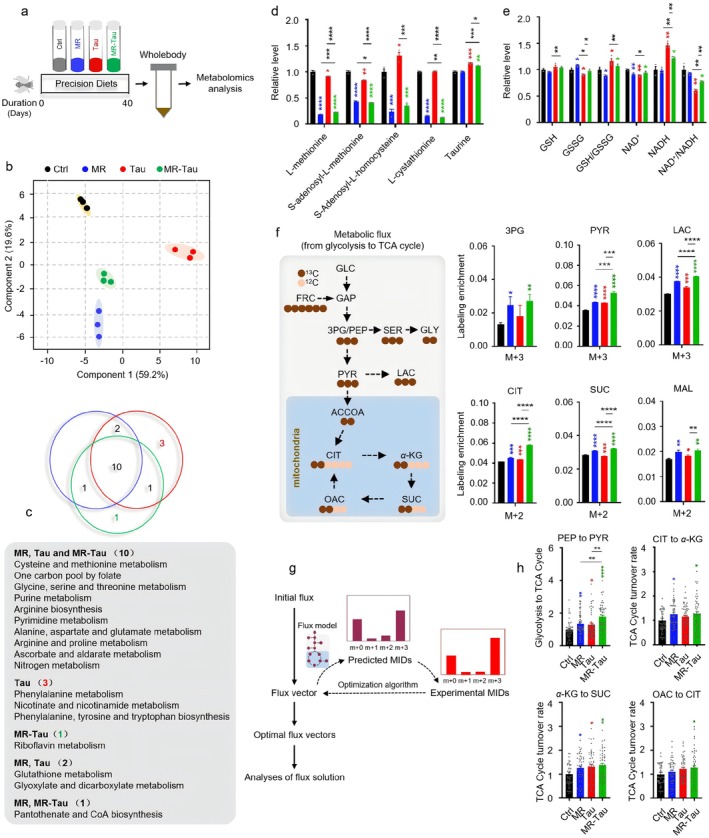
Precision diet reshapes metabolism homeostasis (a) Schematic of the dietary intervention. (b) Principal component analysis (PCA). (c) Pathway enrichment analysis. (d) Relative levels of methionine‐related and taurine‐associated metabolites. (e) Redox balance indicators. (f) Relative labeling enrichment of glycolysis‐TCA cycle metabolites. (g) Experimental design for central carbon metabolic flux analysis. (h) Quantitative central carbon metabolic flux analysis. Unless otherwise indicated, *n* = 10 flies per group. All experiments were independently repeated three times. Survival‐independent comparisons were evaluated using unpaired two‐tailed *t*‐tests unless otherwise specified. Data are presented as mean ± s.e.m. Flux values were estimated by computational optimization using 10,000 simulated replicates. Final flux estimates represent the mean of the 50 simulations with the smallest residual error relative to target metabolite labeling patterns. Color‐coded asterisks indicate statistical significance relative to control for MR (blue), Tau (red), and MR + Tau (green) groups: *p < 0.05, **p < 0.01, ***p < 0.001, ****p < 0.0001. α‐KG, α‐ketoglutarate; 3PG, 3‐phosphoglycerate; ACCOA, acetyl coenzyme A; CIT, citrate; FRC, fructose; GAP, glyceraldehyde‐3‐phosphate; GLC, glucose; GLY, glycine; GSH, glutathione; GSSG, glutathione disulfide; LAC, lactate; MAL, malate; MID, mass isotopomer distribution; NAD+, nicotinamide adenine dinucleotide; NADH, nicotinamide adenine dinucleotide hydrogen; OAC, oxaloacetate; PEP, phosphoenolpyruvate; PYR, pyruvate; SER, serine; SUC, succinate.

**FIGURE A3:**
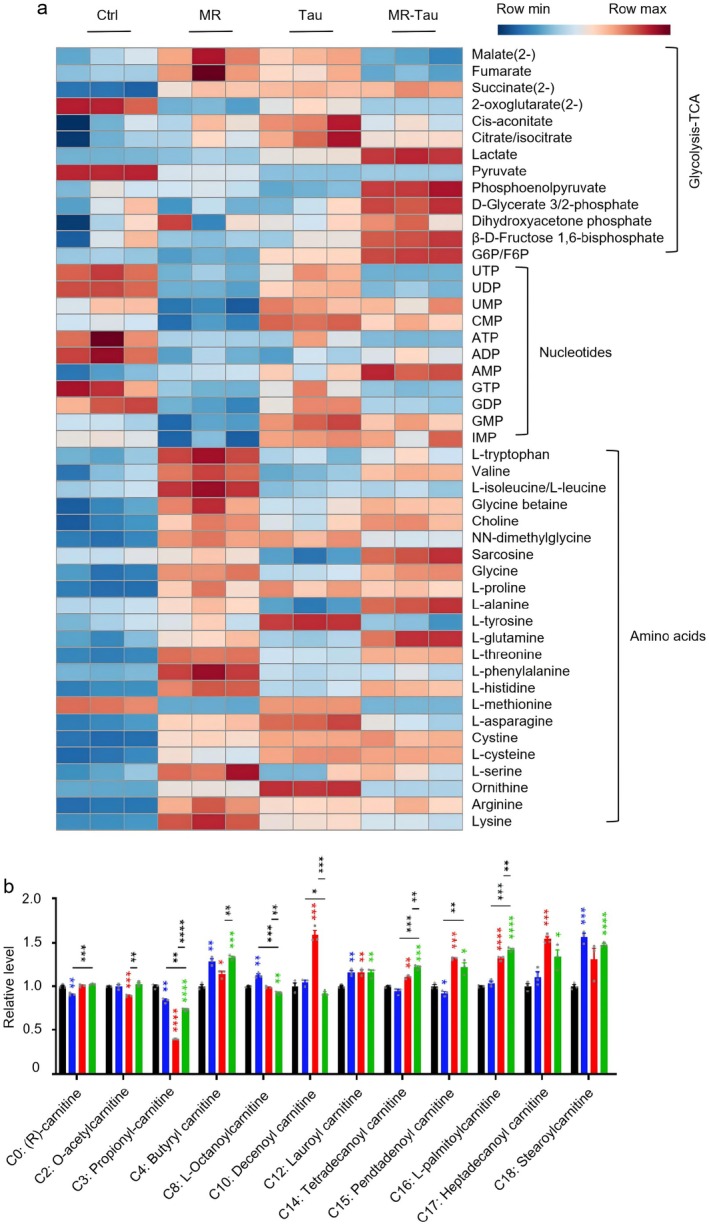
Precision diet modulates carnitine and central carbon metabolism. (a) Heatmap. (b) Relative levels of carnitine and acylcarnitine species. Blue, red, and green asterisks indicate statistically significant differences in the MR, Tau, and MR‐Tau groups, respectively, compared with the control group. *n* = 10 flies per group. All experiments were independently repeated three times. Data are presented as mean ± s.e.m. Statistical significance was determined by unpaired, two‐tailed Student's *t*‐tests unless otherwise specified. *p < 0.05, **p < 0.01, ***p < 0.001, ****p < 0.0001.

To investigate how precision diets influence systemic metabolic homeostasis, we performed targeted metabolomic analysis on whole‐body tissues from flies subjected to each dietary condition (Figure 2a). Metabolite profiles were generated using liquid chromatography coupled with high‐resolution mass spectrometry (LC–MS). Principal component analysis (PCA) indicated distinct metabolic profiles for the different groups (Figure 2b), suggesting that these precision diets induce broad metabolic reprogramming. Notably, the MR‐Tau exhibited coordinated and pronounced changes in nucleotide, amino acid, and mitochondrial metabolites (Figure A3a), consistent with an additive impact on systemic metabolism.

Pathway enrichment analysis identified 10 pathways commonly altered across all interventions, including one‐carbon metabolism (methionine and cysteine metabolism, folate cycle), amino acid metabolism (glycine, serine, and threonine metabolism; arginine and proline metabolism), and purine metabolism (Figure 2c). Consistently, the quantification of methionine‐ and taurine‐related metabolites from these pathways revealed significant changes across interventions (Figure 2d). Remarkably, MR‐Tau uniquely affected pathways related to glutathione biosynthesis and mitochondrial redox regulation (Figure 2e), suggesting altered antioxidant capacity and mitochondrial function.

Carnitine plays an essential role in transporting long‐chain fatty acids into mitochondria, thereby supporting β‐oxidation and cellular energy metabolism (Longo et al. 2016; Reuter and Evans 2012; Virmani and Cirulli 2022). Age‐associated declines in carnitine metabolism impair mitochondrial function and lipid utilization (Hagen et al. 2002; Noland et al. 2009), whereas supplementation has been shown to improve metabolic health in aging (Hagen et al. 2002). In our metabolomic analysis, MR‐Tau led to a marked accumulation of carnitine‐related metabolites (Figure A3b) together with enrichment of the endogenous carnitine biosynthesis pathway (Figure 2c). These findings are consistent with enhanced mitochondrial fatty‐acid transport and oxidative metabolism, which may contribute to maintaining metabolic integrity during aging.

To further assess the effects of different dietary conditions on mitochondrial function and redox balance, we performed stable isotope tracing. This analysis suggested that MR‐Tau markedly enhanced TCA cycle flux (Figure 2f–h), accompanied by increased production of one‐carbon donors and improved redox homeostasis: metabolic hallmarks of resilience associated with healthy aging.

### 2.3 MR‐Tau Intervention Modulates Gut Mitochondrial Metabolism and Supports Systemic Metabolic Homeostasis

**FIGURE A4:**
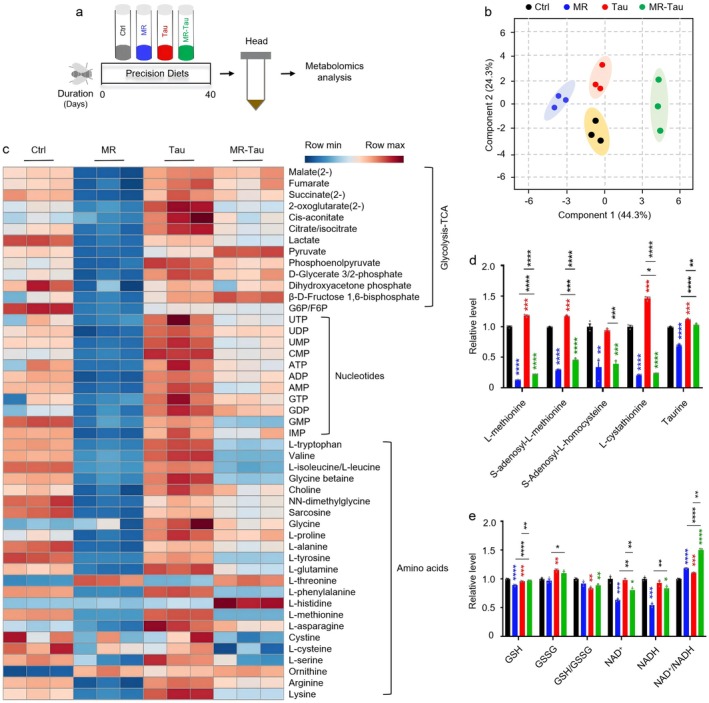
Precision diet reshapes head metabolic landscape. (a) Experimental design. (b) Principal component analysis (PCA). (c) Heatmap. (d) Relative levels of methionine‐related and taurine‐related metabolites. (e) Redox balance. Blue, red, and green asterisks indicate statistical comparisons of MR, Tau, and MR‐Tau groups versus the control group, respectively. *n* = 30. All experiments were independently repeated three times. Data are presented as mean ± s.e.m., *p* values were obtained by unpaired, two‐tailed *t*‐tests unless otherwise specified. *p < 0.05, **p < 0.01, ***p < 0.001, ****p < 0.0001.

The MR‐Tau combination significantly reduced food intake (Figure 1f) without affecting body weight (Figure A2). Given the central regulation of feeding behavior (Kim et al. 2021; Ribeiro and Dickson 2010), we first examined the impact of precision diets on brain metabolism. Metabolomic profiling of fly heads suggested distinct signatures across dietary conditions (Figure A4a,b), with MR generally associated with reduced levels of brain metabolites, whereas Tau or MR‐Tau tended to preserve or elevate these levels (Figure A4c). MR markedly depleted methionine‐related metabolites, whereas taurine supplementation elevated methionine cycle intermediates and partially restored MR‐induced reductions (Figure A4d). Notably, MR‐Tau markedly increased whole‐body mitochondrial TCA flux (Figure 2f,h) and elevated the pyruvate/lactate ratio in heads (Figure A4e), suggesting enhanced systemic oxidative metabolism and a coordinated additive effect on brain energy metabolism and feeding regulation.

**FIGURE 3:**
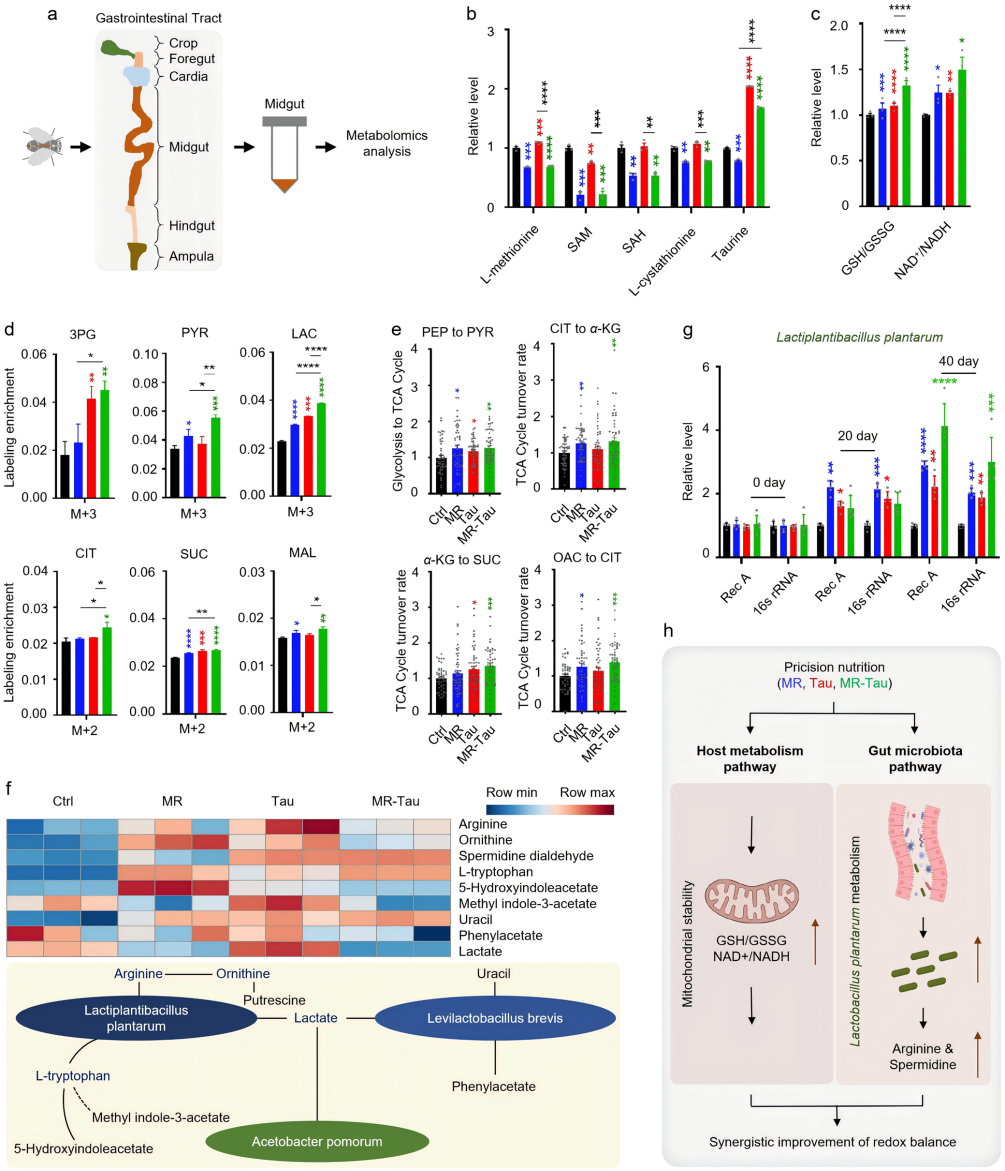
Precision nutrition regulates gut and microbial metabolic homeostasis. (a) Schematic of the experimental design. (b) Relative levels of methionine‐related and taurine‐associated metabolites in the gut. (c) Redox balance indicators. (d) Relative labeling enrichment of glycolysis‐TCA cycle metabolites. (e) Quantitative central carbon metabolic flux analysis. (f) Metabolic network of gut microbiota‐associated pathways. (g) Effect of precision diet on gut *Lactiplantibacillus plantarum* abundance. (h) Proposed model summarizing the effects of precision nutrition on host‐microbiotametabolic homeostasis. Unless otherwise indicated, *n* = 30 biological replicates per group. All experiments were independently repeated three times. For panel (h), schematic elements were derived from experimental findings; quantitative measurements were performed with five technical replicates where applicable. Flux values were estimated by computational optimization using 10,000 simulated replicates. Final flux estimates represent the mean of the 50 simulations with the smallest residual error relative to target metabolite labeling patterns. Data are presented as mean ± s.e.m. Statistical comparisons were performed using unpaired two‐tailed *t*‐tests unless otherwise specified. Color‐coded asterisks indicate statistical significance relative to control for MR (blue), Tau (red), and MR + Tau (green) groups: *p < 0.05, **p < 0.01, ***p < 0.001, ****p < 0.0001. Abbreviations: SAM, S‐adenosyl‐L‐methionine; SAH, S‐adenosyl‐L‐homocysteine.

**FIGURE A5:**
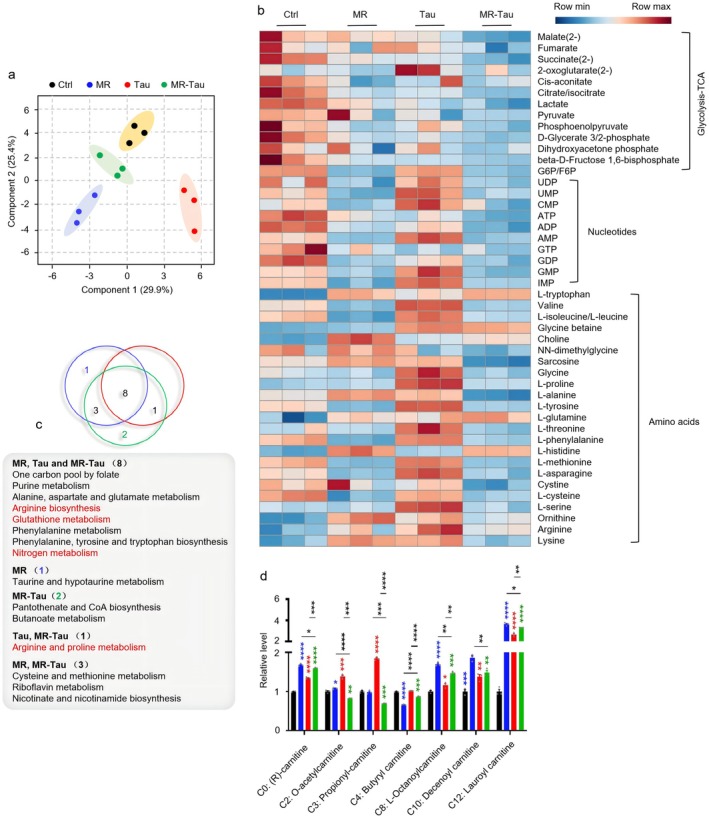
Precision nutrition regulates gut and microbial metabolic homeostasis. (a) PCA. (b) Pathway enrichment analysis. (c) Heatmap. (d) Relative levels of carnitine and acylcarnitine species. *n* = 30. All experiments were independently repeated three times. Data are presented as mean ± s.e.m., *p* values were obtained by unpaired, two‐tailed *t*‐tests unless otherwise specified. *p < 0.05, **p < 0.01, ***p < 0.001, ****p < 0.0001.

Given the gut's pivotal role in nutrient sensing (Kennedy et al. 2025; Liang et al. 2017) and its close association with host metabolism and the microbiota (Guo et al. 2015; Storelli et al. 2011), we next evaluated how MR, Tau, and MR‐Tau influence gut metabolic homeostasis. Following established protocols (Lemaitre and Aliaga 2013), we focused on the midgut (Figure 3a), the primary site of nutrient absorption and functionally analogous to the mammalian small intestine (Buchon, Osman, et al. 2013). Beyond digestion, the midgut contributes to immunity and systemic metabolism via dynamic host‐microbe interactions (Miguel‐Aliaga et al. 2018; Buchon, Osman, et al. 2013; Lemaitre and Aliaga 2013). Targeted metabolomic profiling revealed distinct dietary signatures (Figure A5a), with remodeling in nucleotides, amino acids, and intermediary metabolites (Figure A5b). Pathways associated with methionine and taurine metabolism (Figure 3b and Figure A5c), redox homeostasis (Figure 3c), and carnitine metabolism (Figure A5d) were notably changed, with the most pronounced alterations in the MR‐Tau group. These changes align with previous findings linking improved antioxidant capacity and mitochondrial function to increased longevity (Wei et al. 2024; Xu et al. 2013; Yaku et al. 2018; Zhang et al. 2016).

TCA cycle flux was increased in the midguts of MR‐Tau flies (Figure 3d,e), indicative of enhanced mitochondrial function. Pathway enrichment analysis further identified eight metabolic pathways consistently altered across all interventions (Figure A5c), including one‐carbon metabolism and multiple amino acid‐related pathways. Taken together, these results suggest that MR‐Tau is associated with enhanced gut mitochondrial activity and one‐carbon metabolism, which may contribute to improved redox balance and systemic metabolic stability—features often linked to healthy aging. Notably, although MR and Tau reduced food intake, the extent to which these gut metabolic changes are separable from altered feeding behavior remains to be determined.

### 2.4 MR‐Tau Enhances Microbial Metabolic Activity and Supports Gut Homeostasis

Gut microbiota are integral to host metabolic regulation, immune function, and redox balance, thereby influencing healthspan and aging (Clark et al. 2015; Fung et al. 2025; Montanan et al. 2024; Saravi et al. 2025; Schretter et al. 2018; Song et al. 2023; Storelli et al. 2018). Among the evolutionarily conserved commensals shared between *Drosophila* and mammals are *Lactiplantibacillus plantarum* and *Levilactobacillus brevis*, which serve functionally analogous roles across species (Clark et al. 2015; Guo et al. 2015; Lemaitre and Aliaga 2013; Montanan et al. 2024; Storelli et al. 2011). In contrast, *Acetobacter pomorum* , a dominant fly‐specific symbiont, is well characterized for its effects on host development and metabolic regulation (Montanan et al. 2024). Several microbial taxa are linked to specific metabolic pathways and their associated metabolites: *L. plantarum* is involved in amino acid catabolism and serotonin/indole signaling pathways, producing metabolites such as arginine (Bringel and Hubert 2003; Kim et al. 2023), tryptophan (Hou et al. 2023; Xu et al. 2025), and indole derivatives (Wang et al. 2023; Zhou et al. 2022); *L. brevis* (Lee et al. 2013; Saravi et al. 2025; Sridharan et al. 2014) participates in nucleotide and aromatic amino acid metabolism, generating uracil and phenylacetic acid; and *A. pomorum* contributes to lactate catabolism, which may modulate host energy balance through central carbon metabolism (Shin et al. 2011).

To assess whether dietary interventions influence microbiota‐associated metabolites, we profiled representative compounds. Taurine supplementation was associated with increased levels of arginine, ornithine, and tryptophan, as well as an enhancement of spermidine metabolism (Figure 3f), consistent with elevated microbial metabolic activity and potentially strengthened host‐microbe interactions (Bringel and Hubert 2003; Kim et al. 2023). These findings align with evidence that tryptophan‐enriched diets promote *Lactobacillus*‐linked longevity benefits (Xu et al. 2025).

**FIGURE A6:**
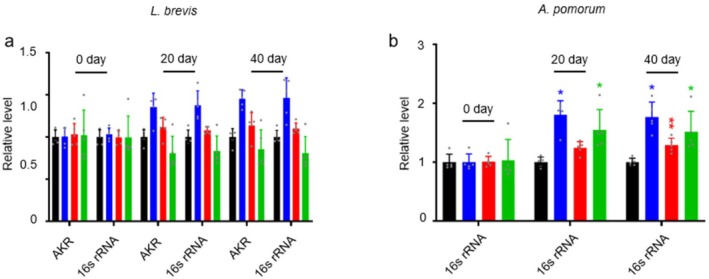
Precision nutrition regulates gut microbial abundance. (a) Relative abundance of *Levilactobacillus brevis* in fly guts under the combined dietary intervention at 0, 20, and 40 days after the start of treatment. Bacterial load was quantified by qPCR using an *L. brevis‐*specific *aldo‐keto reductase* (*AKR*) gene and normalized to total bacterial 16S rRNA levels. (b) Relative abundance of *Acetobacter pomorum* under the same conditions. Relative abundance was calculated using the ΔΔ*Ct* method, with the control group at day 0 set to 1. *n* = 30 biological replicates per group. Microbial quantification was performed using five independent biological replicates per group. Data are presented as mean ± s.e.m.

We next quantified bacterial abundance using conserved molecular markers (*recA* and *16S rRNA*) (Martino et al. 2018; Storelli et al. 2011; Torriani et al. 2001; Turnbaugh et al. 2007; Whitman 2009). Longitudinal analysis revealed a gradual increase in *L. plantarum* abundance, with the most pronounced change in the MR‐Tau group (Figure 3g). *A. pomorum* also showed a moderate rise (Figure A6b), while *L. brevis* remained unchanged (Figure A6a). These observations suggest that *L. plantarum* could contribute to the metabolic effects of MR‐Tau, potentially via arginine biosynthesis and support of host energy and redox balance. Altogether, these results suggest that precision dietary interventions can modulate the gut microbial ecosystem, with MR‐Tau associated with enhanced *L. plantarum* representation and microbial metabolic activity. Such changes may help maintain intestinal functional stability and support systemic metabolic homeostasis, features relevant to healthy aging (Figure 3h).

### 2.5 MR‐Tau Combined With Exercise Supports Lifespan Extension, Physical Performance, and Metabolic Health

**FIGURE 4:**
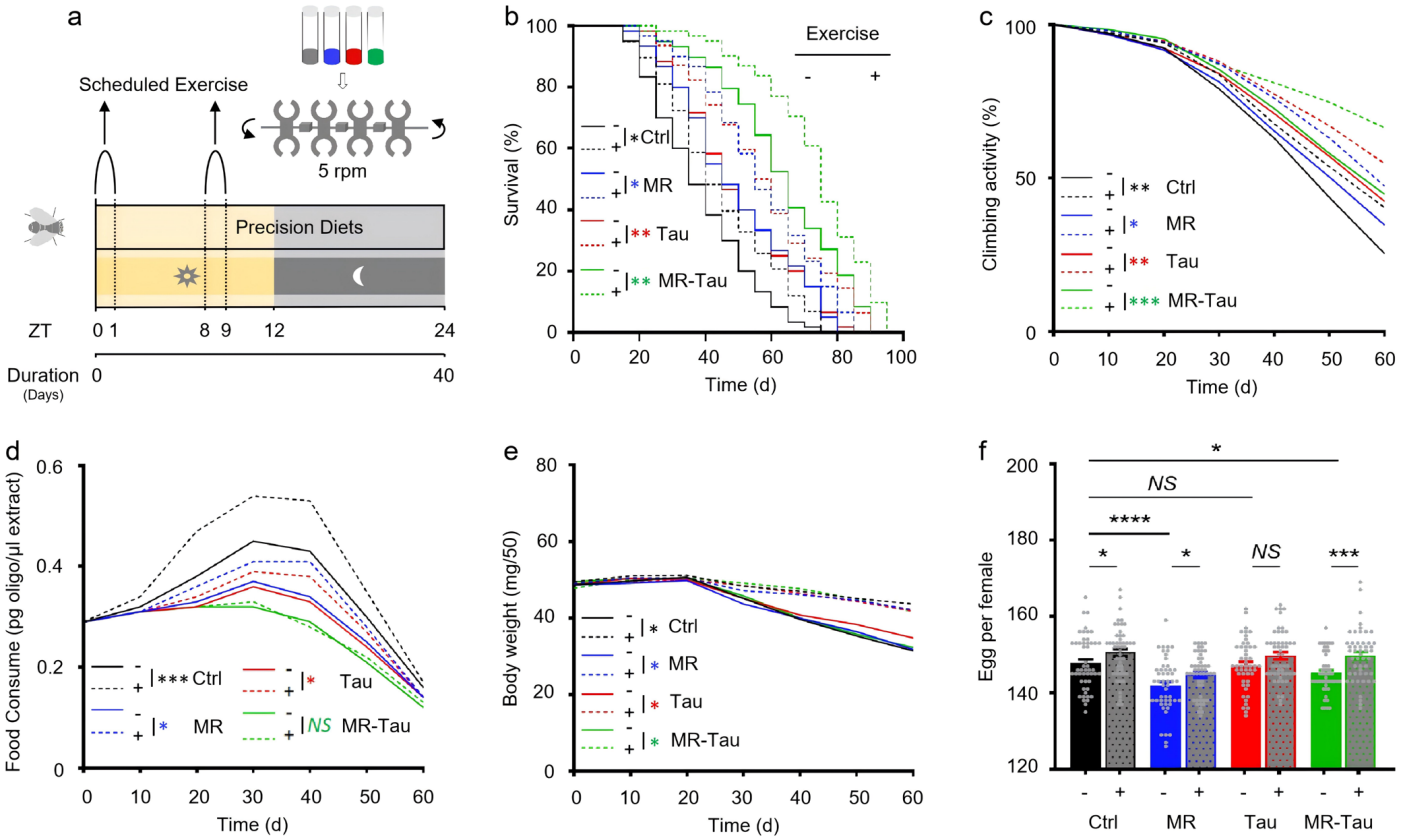
Exercise combined with precision nutrition enhances anti‐aging effects. (a) Schematic of the experimental design. (b) Survival curves under control, exercise, precision nutrition, or combined interventions. (c) Climbing activity. (d) Food consumption. (e) Body weight. (f) Egg production. Lifespan (*n* = 60), climbing (*n* = 50), food consumption (*n* = 50), body weight (*n* = 150) and egg production (*n* = 50) assays were performed per group. Unless otherwise indicated, cohorts consisted of a 1:1 male‐to‐female ratio. Survival assays were conducted in parallel male and female virgin cohorts. For egg production assays (f), experimental females were mated with control‐fed males to standardize paternal dietary effects. Survival curves were analyzed using the log‐rank (Mantel–Cox) test. Other comparisons were performed using unpaired two‐tailed *t*‐tests unless otherwise specified. All experiments were independently repeated three times. Data are presented as mean ± s.e.m. NS, not significant, *p* > 0.05, *p < 0.05, **p < 0.01, ***p < 0.001, ****p < 0.0001.

**FIGURE A7:**
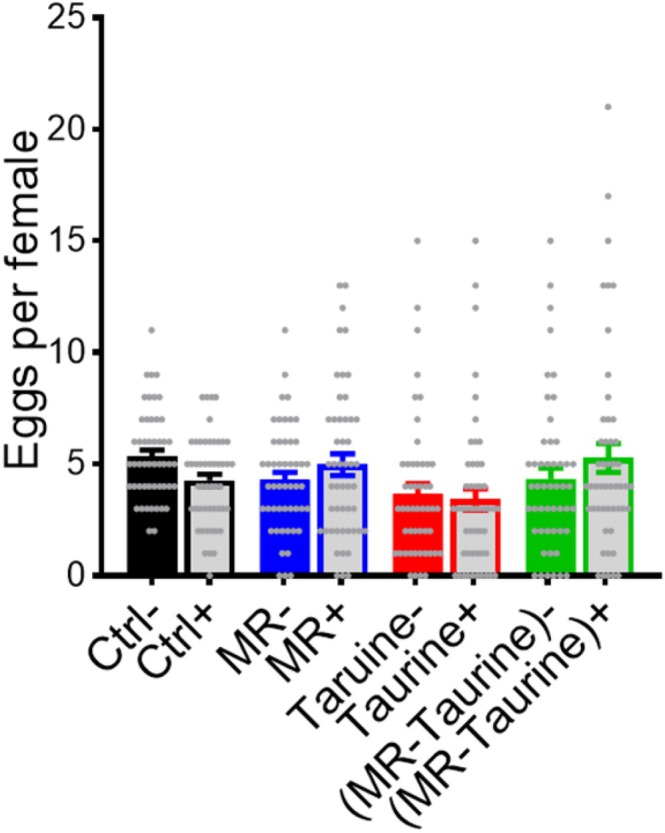
Egg‐laying during late adulthood (days 34–53). Daily egg‐laying per female from day 34 to day 53 is shown for all groups. Each dot represents one female; bars indicate mean ± SEM. Egg production declines sharply in late life, and survival differences between groups (e.g., ~10 flies remaining in Ctrl vs. ~40 in MR‐Tau‐ex at day 50) lead to unequal sample sizes and increased variability. These late‐life data illustrate age‐related reproductive decline and do not affect the conclusions from early‐adulthood cumulative egg counts (days 3–33, main Figure 4f).

Exercise is a well‐established modulator of systemic metabolism and a protective factor against age‐related functional decline (Geng et al. 2025; Janssens et al. 2024; Liu, Hua, and Xin 2024; MoTrPAC, S. G 2025) but its interactions with precision dietary strategies remain insufficiently understood. Building on the metabolic and microbial improvements associated with MR‐Tau, we next investigated whether combining this dietary approach with moderate physical activity could produce additive effects on healthspan. To address this, we implemented a moderate‐intensity exercise regimen across different dietary conditions (Figure 4a). Exercise extended lifespan in all groups, with the most pronounced longevity observed in the MR‐Tau‐exercise cohort (Figure 4b), suggesting a possible additive interaction. Late‐life egg‐laying showed the expected age‐related decline and greater variability due to survival differences, but did not change the conclusions from early‐adulthood reproductive output (Figure A7).

In parallel, longitudinal assessments of locomotor function suggested that all interventions attenuated age‐related decline, with MR‐Tau‐exercise maintaining the highest average physical performance over time (Figure 4c). These findings are consistent with improved neuromuscular preservation under combined intervention (Janssens et al. 2024). Interestingly, although exercise generally increased food intake, MR‐Tau‐fed flies sustained lower feeding rates even during physical activity (Figure 4d), suggesting that diet composition remained a dominant driver of feeding behavior. Body weight was stable across conditions (Figure 4e).

Notably, exercise also partially mitigated the reduced fecundity observed with MR and MR‐Tau (Figure 4f), aligning with previous evidence that nutritional and behavioral interventions can jointly influence reproductive and metabolic outcomes (Locasale 2022). Taken together, these results suggest that combining MR‐Tau with moderate exercise confers broad physiological benefits–including extended lifespan, maintained locomotor capacity, metabolic stability, and partial preservation of reproductive function.

### 2.6 MR‐Tau‐Exercise Remodels the Gut Metabolic Microenvironment Through Mitochondrial‐Microbial Interactions

**FIGURE 5:**
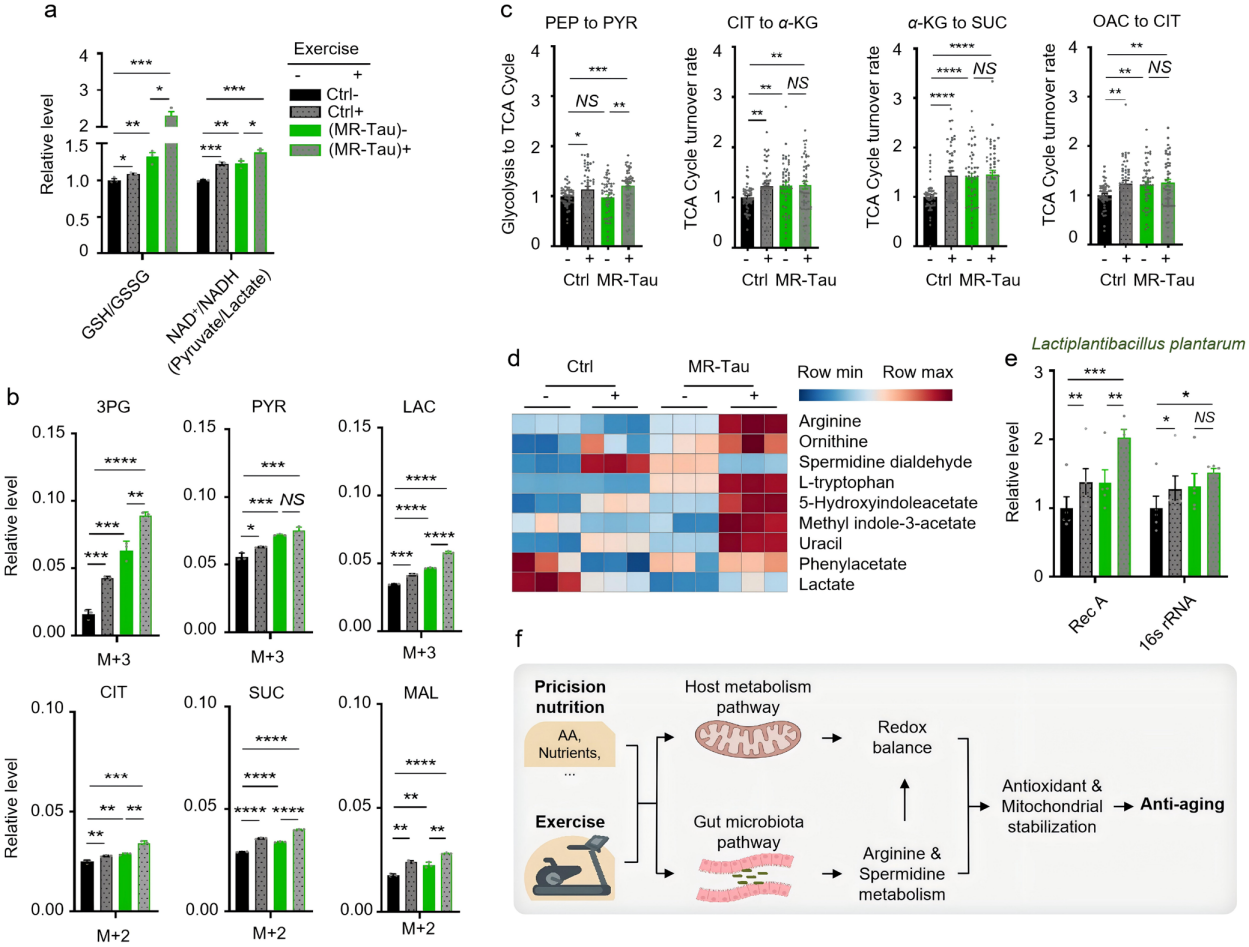
Combined exercise and precision nutrition remodel gut metabolism and reinforce anti‐aging capacity. (a) Redox balance. (b) Relative labeling enrichment of glycolysis‐TCA cycle metabolites. (c) Quantitative central carbon metabolic flux analysis. (d) Relative levels of metabolites associated with gut microbial activity. (e) Effect of combined intervention on gut *Lactiplantibacillus plantarum* abundance. (f) Schematic model summarizing host‐microbiota metabolic remodeling under combined intervention. Unless otherwise indicated, *n* = 30 biological replicates per group. Each biological replicate consisted of pooled adult gut samples. All experiments were independently repeated three times. Fluxes were estimated using metabolic flux analysis (MFA) through optimization of 10,000 simulated replicates; final flux values represent the mean of the 50 simulations with the smallest residual error between simulated and experimentally measured metabolite labeling patterns. Microbial quantification was performed using five independent biological replicates per group. Data are presented as mean ± s.e.m. Statistical comparisons were performed using unpaired two‐tailed *t*‐tests unless otherwise specified. NS, not significant; *p* > 0.05, *p < 0.05, **p < 0.01, ***p < 0.001, ****p < 0.0001.

**FIGURE A8:**
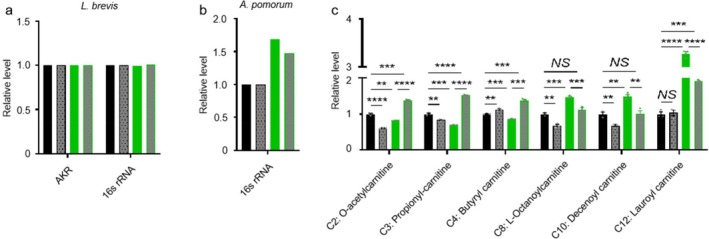
Exercise and precision nutrition additively remodel gut microbial and metabolic profiles. (a) Relative abundance of *Levilactobacillus brevis* in the gut under control conditions, precision nutrition alone, exercise alone, or the combined intervention. Abundance was quantified by qPCR using an *L. brevis‐*specific *aldo‐keto reductase* (*AKR*) gene and normalized to total bacterial 16S rRNA. (b) Relative abundance of *Acetobacter pomorum* . (c) Relative levels of carnitine and acylcarnitine species in flies under the indicated interventions, measured by targeted metabolomics. *n* = 30 biological replicates per group. Each biological replicate consisted of pooled adult flies. For qPCR assays (a, b), each biological replicate was measured in five technical replicates. Data are presented as mean ± s.e.m. NS, not significant *p* > 0.05; **p < 0.01, ***p < 0.001, ****p < 0.0001, *p* values were obtained by unpaired, two‐tailed *t*‐tests unless otherwise specified.

The gut is a central regulator of systemic energy metabolism and redox homeostasis (Turnbaugh et al. 2007). Consistent with prior reports (Locasale 2022; Martino et al. 2018), dietary composition modulated the gut microenvironment through changes in microbial composition and metabolic function (Figure 3f,g, Figures A5 and A6). To investigate how MR‐Tau‐exercise shapes host‐microbe metabolic crosstalk, we assessed redox balance, lipid metabolism, microbial dynamics, and central metabolic fluxes. Redox‐sensitive metabolites increased stepwise from control to MR‐Tau, peaking in the MR‐Tau‐exercise group (Figure 5a), consistent with enhanced redox buffering capacity and mitochondrial function (Wei et al. 2024). This was accompanied by increased expression of *L. plantarum recA* and *16S rRNA* (Figure 5e), while *L. brevis* remained unchanged (Figure A8a), suggesting a species‐specific microbial response.

**FIGURE A9:**
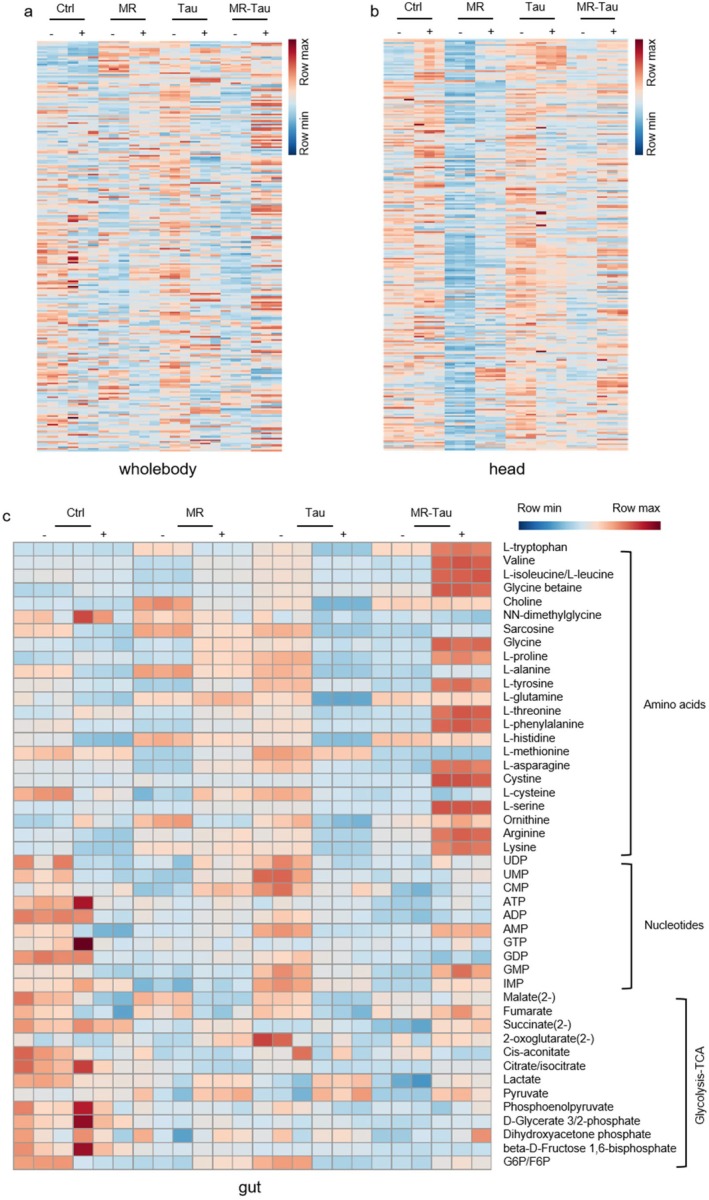
Tissue‐specific metabolic remodeling under combined exercise and precision nutrition intervention. (a–c) Metabolite set enrichment analysis of significantly altered metabolites in whole‐body (a), head (b), and gut (c) tissues under combined exercise and precision nutrition intervention compared to controls. *n* = 10 (whole body) or *n* = 30 (head or gut) biological replicates per group. Each biological replicate consisted of pooled tissues from adult flies. All experiments were independently repeated three times. Data are presented as mean ± s.e.m.

Consistent with elevated mitochondrial lipid utilization, free, short‐, and medium‐chain carnitine levels were increased in MR‐Tau and remained above baseline with exercise (Figure A8c). Stable isotope tracing analysis further indicated that MR‐Tau‐exercise produced the strongest activation of central carbon metabolism among all tested conditions (Figure 5b,c and Figure A9c), supporting additive engagement of mitochondrial energy pathways.

Microbial‐associated metabolites, including *L. plantarum* ‐associated arginine and tryptophan derivatives, were significantly enriched under MR‐Tau‐exercise (Figure 5d), consistent with increased microbiota‐associated metabolic signatures. Interestingly, *A. pomorum* abundance was not altered by exercise alone but was increased in MR‐Tau and remained elevated when exercise was added (Figure A8b), suggesting that diet exerts the primary influence on microbial composition, while exercise may augment diet‐induced changes.

**FIGURE A10:**
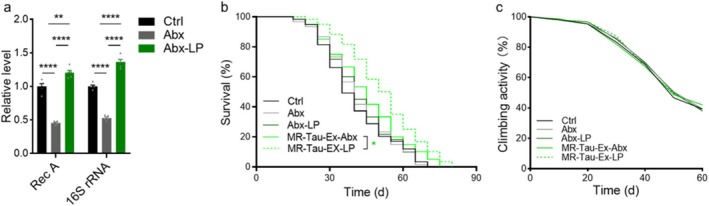
Validation of *L. plantarum* recolonization and its contribution to lifespan under MR‐Tau‐exercise. (a) Relative abundance of *L. plantarum* (LP) in the gut measured by qPCR in control flies, antibiotic‐treated (Abx) flies, and Abx flies recolonized with LP. (b) Survival curves of flies under MR‐Tau‐exercise conditions with antibiotic treatment and LP recolonization. (c) Negative geotaxis assay showing climbing performance across treatment groups. Lifespan assays were conducted in parallel male and female virgin cohorts (*n* = 60 per sex per group). Climbing activity and food consumption assays were performed with *n* = 50 flies per group. All experiments were independently repeated three times. Data are presented as mean ± s.e.m. Statistical significance was determined by unpaired, two‐tailed *t*‐tests unless otherwise specified. Survival curves were analyzed using the log‐rank (Mantel–Cox) test. Blue, red, and green asterisks indicate significant differences for MR, Tau, and MR + Tau groups compared with control, respectively: *p < 0.05, **p < 0.01, ****p < 0.0001.

These alterations were consistently observed across whole‐body, gut, and brain metabolomes (Figure A9), suggesting that MR‐Tau‐exercise integrates host and microbial metabolic responses across multiple tissues. Consistent with a role for the microbiota, *L. plantarum* recolonization was successfully established and partially restored the lifespan benefits of MR‐Tau‐exercise without affecting locomotor performance (Figure A10). Overall, these findings are consistent with the interpretation that this combined intervention reconfigures the gut metabolic niche; however, direct causal evidence for each proposed node awaits future studies: enhancing redox capacity, supporting lipid utilization, and modulating microbial function in a manner that aligns with improved metabolic homeostasis and functional health.

## 3 Discussion

Our study suggests that MR‐Tau‐exercise robustly extends lifespan in *D. melanogaster* while preserving locomotor performance, reproductive output, and metabolic homeostasis. Mechanistically, this multimodal intervention enhances gut mitochondrial redox balance and central carbon metabolism, accompanied by selective enrichment of *L. plantarum* (Figure 5f). These coordinated adaptations are consistent with a working model of a gut‐mitochondrial axis that warrants further investigation, in which dietary and behavioral inputs reshape the intestinal metabolic microenvironment to support systemic resilience.

Dietary restriction strategies such as MR or caloric restriction extend lifespan across species but often involve functional trade‐offs, including reduced fertility or impaired physical performance (Gao et al. 2019; Grandison et al. 2009; Lee et al. 2014; Redman and Ravussin 2011; Wei et al. 2024). In contrast, the MR‐Tau‐exercise combination mitigates these trade‐offs. Enhanced gut antioxidant capacity and increased TCA cycle flux suggest strengthened mitochondrial buffering and oxidative metabolism (Figure 5). Concurrent enrichment of microbiota‐associated metabolites, including arginine and tryptophan derivatives linked to *L. plantarum* , is consistent with a potential role for microbial‐host metabolic crosstalk in supporting mitochondrial function. While these associations align with prior reports implicating *Lactiplantibacillus* species in redox regulation and longevity, definitive attribution of specific metabolites to microbial production will require germ‐free models, microbial transplantation, or targeted microbial genetic perturbation.

Importantly, recolonization experiments provide evidence that *L. plantarum* partially mediates the lifespan benefits of MR‐Tau‐exercise, yet the incomplete rescue coupled with the lack of restored locomotor performance suggests that other microbial taxa or host pathways are also involved, and that microbial mediation is partial rather than exclusive. These findings refine the mechanistic model: mitochondrial remodeling represents a central adaptive response, with microbiota‐dependent inputs modulating but not solely determining the systemic phenotype.

Tissue‐specific metabolomic further suggested coordinated inter‐organ remodeling. In the gut, pathways related to energy production and redox balance were upregulated (Figure 3c–e and Figure A5), while in the brain, TCA cycle activity and neuroactive metabolites were restored (Figure A4c). This coordinated metabolic restructuring suggests that intestinal mitochondrial adaptation may influence distal tissues through metabolite‐mediated communication. Future studies integrating inter‐organ flux tracing, microbial genetics, and behavioral analyses will be necessary to dissect how diet‐exercise‐microbiota interactions shape gut‐brain signaling and organismal resilience during aging.

Although these findings arise from a *Drosophila* model, several independent lines of evidence support translational relevance. MR extends lifespan in rodents, taurine supplementation improves metabolic health in mammals, and exercise enhances mitochondrial function across species. Whether their additive effects converge on comparable gut‐mitochondrial mechanisms in mammals remains to be tested. Rigorous validation using tissue‐resolved metabolomics and community‐level microbiome profiling in murine systems will be essential to evaluate efficacy, safety, and tissue specificity in more complex organisms.

The partial preservation of egg‐laying capacity under MR‐Tau‐exercise, despite significant lifespan extension, is notable given the well‐established tradeoff between reproduction and longevity under dietary restriction in *Drosophila*. Typically, dietary restriction extends lifespan at the expense of reduced reproductive output, reflecting resource allocation tradeoffs. Our observation that MR‐Tau‐exercise attenuated this tradeoff‐extending lifespan while maintaining partially preserved fecundity‐suggests that the combination intervention may enhance metabolic efficiency or nutrient repartitioning. One possible explanation is that enhanced gut mitochondrial redox capacity and TCA cycle flux (Figure 5b,c) improve energy utilization efficiency, allowing allocation of resources to both somatic maintenance and reproduction. Alternatively, taurine's known role in protecting ovarian mitochondrial function, together with exercise‐induced improvements in systemic metabolic flexibility, may collectively offset the reproductive cost typically imposed by MR. Additionally, the selective enrichment of *L. plantarum* and its associated metabolites (e.g., arginine and tryptophan derivatives) could influence reproductive physiology through host‐microbe metabolic crosstalk. While these explanations remain speculative, they provide a framework for future mechanistic dissection, for example by measuring ATP allocation, lipid droplet dynamics in the fat body and ovaries, or targeted genetic manipulation of insulin/IGF signaling pathways.

Thus, rather than providing a complete mechanistic dissection, this study proposes a conceptual framework linking precision diet, exercise, microbial remodeling, and mitochondrial redox metabolism that can guide future hypothesis‐driven investigations.

### 3.1 Limitations and Caveats

Although MR and taurine supplementation (Tau) individually reduced food intake, and the MR‐Tau combination produced the greatest suppression, we cannot exclude the possibility that changes in food palatability or aversive responses rather than purely metabolic adaptation contributed to the reduced feeding. In *Drosophila* dietary restriction paradigms, compensatory feeding is often observed depending on amino acid balance; however, we did not detect such compensation under MR or MR‐Tau, suggesting that these diets may actively suppress appetite rather than simply limit nutrient availability. Nevertheless, altered ingestion behavior itself could partially contribute to the observed lifespan and redox phenotypes. Thus, while our metabolomic and flux analyses suggested gut mitochondrial remodeling, the extent to which these changes are fully separable from feeding behavior remains to be determined. Future studies using pair‐feeding or automated feeding monitors will help dissect the relative contributions of nutrient composition versus caloric intake.

The midgut of *D. melanogaster* is not metabolically uniform; it comprises functionally and chemically distinct regions, including the acidic copper cell region (Cu region), the enteroendocrine‐rich region, and the posterior midgut. Our metabolomic analyses were performed on whole midgut tissue, which inevitably integrates signals from these specialized compartments. Consequently, regional metabolic specialization—particularly the acidic environment of the Cu region, which profoundly influences microbial colonization and metabolism‐may modulate the microbiota‐associated metabolic signatures we observed. Future studies employing region‐specific dissection (e.g., using genetic drivers or microdissection) will be required to resolve spatial metabolic heterogeneity and its contribution to host‐microbe crosstalk.

Several limitations should be considered when interpreting these findings. First, although increased GSH/GSSG and NAD+/NADH ratios suggest enhanced redox buffering and oxidative metabolism, complementary measures such as NADP/NADPH or lactate/pyruvate ratios were not assessed, and redox remodeling should therefore be interpreted cautiously.

Second, multiple metabolites identified by targeted metabolomics are microbiota‐associated, and their precise origin (host versus microbial) cannot be definitively assigned. While recolonization experiments support a functional contribution of *L. plantarum* , they do not allow direct attribution of individual metabolites to microbial synthesis, nor exclude minor contributions from other taxa or host pathways.

Third, microbial profiling relied primarily on targeted qPCR of selected taxa. Community‐level approaches such as 16S rRNA sequencing or shallow metagenomics would provide a more comprehensive ecological context and may uncover additional contributors to metabolic remodeling.

Finally, although the *Drosophila* midgut shares functional features with the mammalian intestine, it is not a direct anatomical or immunological equivalent. Species‐specific differences must therefore be considered when extrapolating mechanistic conclusions.

In summary, despite being derived from lifespan, metabolic flux, and microbiota datasets, our findings are consistent with a gut‐mitochondrial axis in which MR‐Tau‐exercise is associated with enhanced redox buffering and mitochondrial energy metabolism, with *L. plantarum* contributing to part of this adaptive remodeling. Consistent with the possibility that lifespan extension can be uncoupled from functional decline, this work provides a conceptual foundation for integrated dietary and behavioral strategies aimed at promoting systemic resilience during aging.

## 4 Methods

### 4.1 Fly Stocks and Maintenance

*Drosophila melanogaster* (*w* ^1118^) flies were obtained from the Bloomington Stock Center and maintained at 25°C with 60% humidity under a 12:12 h light–dark cycle. Newly eclosed flies (≤ 8 h) were collected, grouped (25 per vial), and transferred to fresh food every 2–3 days.

### 4.2 Diet

Flies were maintained on a CDD adapted from published protocols (Gu et al. 2022; Piper et al. 2014; Piper et al. 2017; Troen et al. 2007; Wei et al. 2024). Modifications to the CDD included changes in agar type, sucrose concentration, nucleoside composition, and amino acid ratios to reflect the *Drosophila* exome (Gu et al. 2022; Piper et al. 2017; Wei et al. 2024). The full formulation of the CDD is provided in Supporting Information 1. Variants of the CDD were prepared for specific experimental needs, such as methionine‐restricted, nutrient‐enriched, or isotope‐labeled diets (U‐^13^C_6_‐Sucrose (fructose), Cambridge, Cat. CLM‐9811‐PK), with the fructose labeling rate shown in Supporting Information 2. Diets were stored at 4°C. For reproductive assays, experimental diets were provided to one sex while the other remained on control food.

### 4.3 Lifespan Assay

Flies were collected within 8 h post‐eclosion, sorted under CO_2_, and randomly allocated to different diets (each group contained three replicates with 10 flies per vial). They were transferred to fresh food every 2–3 days, and deaths were recorded daily. Males and females were assayed in separate, virgin cohorts. All flies used in lifespan analysis were kept virgin to exclude mating‐related effects.

### 4.4 Food Intake Assay With DNA Oligomer Incorporation and qPCR

Three DNA oligomers were prepared in ddH_2_O at final concentrations of 1.75, 2.5, and 3.5 μg/μL, respectively, based on previous studies (Park et al. 2018; Parkhitko et al. 2021). A total of 90 μL of the mixture was applied evenly onto the surface of fly food and air‐dried. Sex‐separated flies were transferred to the food‐containing vials and maintained at 25°C overnight. The following day, flies were briefly anesthetized with CO_2_, and 10 flies per sample were collected for analysis.

For DNA barcode qPCR, flies were homogenized in 70 μL squishing buffer (10 mM Tris–HCl pH 8.2, 25 mM NaCl, 1 mM EDTA, 200 μg/mL proteinase K) using 0.5 mm zirconium beads. Homogenates were incubated at 37°C for 40 min, followed by heat inactivation at 95°C for 5 min. Samples were centrifuged at 10,000 g for 10 min, and 10 μL of the supernatant was used for qPCR using Power SYBR Green PCR Master Mix (Thermo Fisher, Cat. 4367659) on a Viia 7 Real‐Time PCR System (Roche). Amplification was performed at 60°C for 40 cycles.

The sequences of the DNA oligomers and qPCR primers used are as follows:

*DNA Oligomer 1*:

5′‐ACCTACACGCTGCGCAACCGAGTCATGCCAATATAAGCAGATTAGCATTACTTTGAGCAACGTATCGGCGATCAGTTCGCCAGCAGTTGTAATGAGCCCC‐3′.

Forward qPCR Primer 1:5′‐GCAACCGAGTCATGCCAATA‐3′.

Reverse qPCR Primer 1:5′‐TTACAACTGCTGGCGAACTG‐3′.

*DNA Oligomer 2*:

5′‐GGGCAGCAGGATAACTCGAATGTCTTAGTGCTAGAGGCTTGGGGCGTGTAAGTGTATCGAAGAAGTTCGTGTTAAACGCTTTGGAATGACTGTAATGTAG‐3′.

Forward qPCR Primer 2: 5′‐CAGCAGGATAACTCGAATGTCTTA‐3′.

Reverse qPCR Primer 2: 5′‐CAGTCATTCCAAAGCGTTTAACA‐3′.

DNA Oligomer 3:

5′‐CTGTAGTATTCGTCCGACGTTCCTCTCTGCTCGGGTACGCGACGAAGGCTCTACTGGCAGTCGAGATTATCGTACAATTTAGTTCGGCCAACCTGAAGCT‐3′.

Forward qPCR Primer 3:5′‐CTGTAGTATTCGTCCGACGT‐3′.

Reverse qPCR Primer 3:5′‐AGCTTCAGGTTGGCCGAACT‐3′.

### 4.5 Egg Counting

To assess reproductive output, mated females were transferred to fresh diet vials daily. Egg production was quantified from day 3 post‐mating over a 30‐day period. For reproductive assays, only one sex (male or female) was exposed to the experimental diet, while the mating partner remained on control food. Mating was conducted for 5 h on control media (Sepil et al. 2020; Wei et al. 2024). Flies used for egg counting were allowed to mate only once.

### 4.6 Climbing Assay and Body Weight Measurement

Climbing ability was assessed as previously described (Ulgherait et al. 2021; Wei et al. 2024). Briefly, 10 single‐sex flies were transferred into a 23 × 95 mm empty vial, tapped down three times, and the number of flies reaching the top within 20 s was recorded. Three vials per group were tested under each condition. Tests were performed every 10 days over a 2‐month period.

For body weight, 50 flies were collected, weighed every 10 days, and followed up for 2 months. Each experiment was performed on at least 150 flies (3 vials of 50 flies per condition) and repeated 3 times.

### 4.7 *Drosophila* Exercise System

Based on previous studies (Mendez et al. 2016), a TreadWheel apparatus was used for exercise training in *Drosophila*. Each device accommodates 12 fly vials mounted on four axles and fits within a standard *Drosophila* incubator. Vials were rotated lengthwise at a constant speed of 5 rpm by an electric motor, continuously altering the vertical orientation of the vial. This gentle, sustained rotation exploits the flies' innate negative geotaxis, providing a consistent climbing stimulus throughout the training period.

### 4.8 Tissue Preparation

Flies were anesthetized with CO_2_ first. Whole‐body samples were prepared using 10 flies per group, snap‐frozen in liquid nitrogen before storage at −80°C.

For head samples, heads were rapidly dissected under a stereomicroscope using a scalpel. Dissected heads were immediately transferred into Eppendorf tubes and snap‐frozen in liquid nitrogen before storage at −80°C. Each head sample group consisted of heads from 30 flies.

For midgut samples, the digestive system was dissected following established (Miguel‐Aliaga et al. 2018; Buchon, Broderick, and Lemaitre 2013). The midgut was isolated, quickly transferred into Eppendorf tubes, snap‐frozen in liquid nitrogen, and stored at −80°C. Each midgut sample group consisted of midguts from 30 flies.

### 4.9 RNA Extraction and qPCR Analysis

Total RNA was extracted from dissected *Drosophila* midguts using the QIAwave RNA Mini Kit, Cat. 74536 following established protocols (Arribas et al. 2024). To remove contaminating genomic DNA, RNA samples were treated with RQ1 RNase‐Free DNase (Promega, Cat. M6101). cDNA was synthesized from total RNA using the iScript cDNA synthesis kit (Bio‐Rad, Cat. 170‐8891).

Quantitative PCR (qPCR) was performed using Power SYBR Green PCR Master Mix (Thermo Fisher, Cat. 4367659) on a Viia 7 Real‐Time PCR System (Roche). We focused on three representative bacterial species commonly found in the *Drosophila* midgut: *L. plantarum*, *Levilactobacillus brevis*, and *Acetobacter pomorum* .

Gene‐specific primers targeting *16S rRNA*, *recA*, and *aldo/keto reductase* (AKR, *L. brevis* ‐specific) genes were designed based on published sequences (Fusco et al. 2016; Matsuda et al. 2009; Packey et al. 2013; Torija et al. 2010; Tsai et al. 2010). Total bacterial 16S rRNA served as an internal control for normalization (Muyzer et al. 1993). Primer sequences are listed below.

#### 4.9.1 Lactiplantibacillus Plantarum

*16S rRNA* (Packey et al. 2013): Lp‐16S‐Forward 5′‐GTGSTGCAYGGYTGTCGTCA‐3′; Lp‐16S‐Reverse 5′‐ACGTCRTCCMCACCTTCCTC‐3′.

*recA* (Tsai et al. 2010): Lp‐recA‐Forward 5′‐CCGTTTCTGCGGAACACCTA‐3′; Lp‐recA‐Reverse 5′‐TCGGGATTACCAAACATCAC‐3′.

#### 4.9.2 Levilactobacillus Brevis

*16S rRNA* (Matsuda et al. 2009): Lb‐Forward 5′‐ATTTTGTTTGAAAGGTGGCTTCGG‐3′; Lb‐Reverse 5′‐ACCCTTGAACAGTTACTCTCAAAGG‐3′.

*AKR* (Fusco et al. 2016): Lb‐AKR‐Forward 5′‐AATTGATTTTCATACCGCAGAA‐3′; Lb‐AKR‐Reverse 5′‐TTGGCACCGCATGATGTG‐3′.

#### 4.9.3 *Acetobacter pomorum*

*16S rRNA* (Torija et al. 2010): Ap‐16S‐Forward 5′‐TCAAGTCCTCATGGCCCTTATG‐3′; Ap‐16S‐Reverse 5′‐TCGAGTTGCAGAGTGCAATCC‐3′.

#### 4.9.4 Total Bacterial

*16 s rRNA* (Muyzer et al. 1993): 341F‐5′‐ACTCCTACGGGAGGCAGCAG‐3′; 534R‐5′‐ATTACCGCGGCTGCTGG‐3′.

The amplification protocol was as follows: initial denaturation at 95°C for 3 min, followed by 40 cycles of 95°C for 15 s, 55°C for 30 s, and 72°C for 30 s. Melting curve analysis at the end of the run to confirm primer specificity. The relative abundance of bacterial species was calculated using the Δ*Ct* method, normalized to total bacterial *16S rRNA* levels.

### 4.10 Metabolite Extraction

10 flies or 30 flies' heads or midguts were collected under light CO_2_ anesthesia, snap‐frozen in liquid nitrogen, and stored at −80°C. Tissues were ground using a CryoMill, and metabolites were extracted as previously described (Liu et al. 2014). Supernatants were dried in a vacuum concentrator at room temperature and reconstituted in 30 μL of solvent (15 μL water followed by 15 μL methanol/acetonitrile, 1:1, v/v). A 3 μL aliquot was analyzed by liquid chromatography coupled with high‐resolution mass spectrometry using water with 5 mM ammonium acetate (pH 6.9) as mobile phase A and 100% acetonitrile as mobile phase B.

Metabolite separation and detection were performed using an Ultimate 3000 UHPLC system (Dionex) coupled to a Q Exactive mass spectrometer (Thermo Scientific). Metabolites were separated at room temperature using a hydrophilic interaction chromatography (HILIC) method with an XBridge Amide column (100 × 2.1 mm, 3.5 μm; Waters). Mobile phase composition and gradient conditions were described previously (Liu et al. 2014). Briefly, the linear gradient of mobile phase B was: 0 min, 85%; 1.5 min, 85%; 5.5 min, 35%; 10 min, 35%; 10.5 min, 35%; 10.6 min, 10%; 12.5 min, 10%; 13.5 min, 85%; and 20 min, 85%. The flow rate was 0.15 mL/min (0–5.5 min), 0.17 mL/min (6.9–10.5 min), 0.3 mL/min (10.6–17.9 min), and 0.15 mL/min (18–20 min). The mass spectrometer was equipped with a heated electrospray ionization (HESI) source. Key parameters were: evaporation temperature, 120°C; sheath gas, 30; auxiliary gas, 10; sweep gas, 3; spray voltage, 3.6 kV (positive mode) or 2.5 kV (negative mode); capillary temperature, 320°C; and S‐lens level, 55. Full MS scans were acquired over m/z 70–900 at 70,000 resolution, with a maximum injection time of 200 ms and automatic gain control (AGC) target of 3 × 10^6^ ions. Customized mass calibration was performed before data acquisition.

### 4.11 Metabolomics Data Processing and Metabolic Flux Analysis

LC–MS peak extraction and integration were performed using Sieve 2.2 software (Thermo Scientific). Integrated peak intensities were used for downstream analysis. Natural isotope abundance correction was applied as previously described (Liu et al. 2016) for isotope tracing studies.

A metabolic network model was constructed covering glycolysis, the TCA cycle, the pentose phosphate pathway, one‐carbon metabolism, and amino acid biosynthesis (Liu, Liu, and Locasale 2024; Wei et al. 2024). To minimize random errors from batch effects, mass isotopomer distributions (MIDs) of metabolites were averaged across biological replicates. The average MID of each metabolite was predicted as the weighted sum of its precursor MIDs, based on presumed generating fluxes. These average MID data are fitted with the following procedure: MIDs of all target metabolites are predicted by averaging the MID of the precursors, weighted by the corresponding generating fluxes with the presumed value:

$${\tilde{M}}_{i}=\frac{\sum_{\forall j}v_{\mathrm{ji}}M_{\mathrm{ji}}}{\sum_{\forall j}v_{\mathrm{ji}}}$$

${\tilde{M}}_{i}$ is the predicted MID vector of metabolite i; $M_{\mathrm{ji}}$ is the MID of metabolite i produced from a substrate j; $v_{\mathrm{ji}}$: the flux from j to i.

If $M_{\mathrm{ji}}$ is still unknown, it can be deduced by the same procedure until MIDs of all precursors are known. Then, the difference between the predicted and experimental MIDs of target metabolites, was evaluated by sum of squared error:

$$L_{i}={\left\|{\tilde{M}}_{i}-M_{i}\right\|}^{2}=\sum_{j}{\left({\tilde{M}}_{i,j}-M_{i,j}\right)}^{2}$$

$L_{i}$ is the difference of target metabolite i. $M_{i,j}$, ${\tilde{M}}_{i,j}$ the element j in vector $M_{i}$ and ${\tilde{M}}_{i}$.

Sum of $L_{i}$ for all target metabolites was regarded as the total difference $L_{\text{total}}$ to minimize by adjusting flux vector $v=\left\{v_{i}\right\}$ that including all fluxes. Therefore, an optimization problem was defined as:

$$\min_{v}L_{\text{total}}\left(v\right),s.t.A\cdot v^{T}=b,0\leq v_{\min}\leq v\leq v_{\max}$$

$A\cdot v^{T}=b$ is the flux balance requirement and other equality constraints; $v_{\min}$, $v_{\max}$: lower and upper bounds for composite vector v. The solution of this optimization problem $v^{*}$ gives a combination of all fluxes in the network model that fits MID data. Method utilized to solve this optimization problem refers to previous publication (Antoniewicz et al. 2007; Kraft 1988). The optimization was repeated 10,000 times, and the 50 solutions with the lowest final $L_{\text{total}}$ were selected as the final solution set to ensure precision, accuracy, and robustness. In line with our previous study using the model (Wei et al. 2024), the method has been further applied and systematically optimized in this study.

### 4.12 Antibiotic Treatment

Adult *Drosophila melanogaster* were collected within 7 days of eclosion and separated by sex. To eliminate the endogenous gut microbiota, flies were reared on standard diet supplemented with a broad spectrum antibiotic cocktail for 5 days. The antibiotic diet contained ampicillin (200 μg/mL), kanamycin (200 μg/mL), tetracycline (50 μg/mL), and erythromycin (300 μg/mL), each dissolved in appropriate solvents and mixed into autoclaved food media (Heys et al. 2018). Control flies were maintained on standard diet without antibiotics. Successful depletion of endogenous bacteria was confirmed by plating fly homogenates and observing undetectable colony‐forming units after 5 days of antibiotic exposure.

### 4.13 *Lactiplantibacillus plantarum* Preparation and Inoculation

*Lactiplantibacillus plantarum* was cultured overnight in de Man, Rogosa and Sharpe (MRS) broth at 37°C. Cells were harvested by centrifugation (4000 × *g*, 10 min), washed twice with sterile phosphate buffered saline (PBS), and resuspended in PBS to an OD600 ≈ 1.0 (≈1 × 10^9^ CFU/mL). Flies previously treated with antibiotics were transferred to sterile vials containing autoclaved food. A volume of 100 μL of the Lp suspension was evenly spread onto the food surface and allowed to air dry under sterile conditions. Flies were allowed to feed on the Lp seeded food for 16–24 h to establish colonization, after which they were transferred to fresh sterile food for the remainder of the experiment (Fast et al. 2018).

### 4.14 Statistics and Reproducibility

All experiments were performed with at least three biological replicates and were independently repeated two to three times. Data are presented as mean ± s.e.m. unless otherwise indicated. Statistical significance was determined using unpaired two‐tailed *t*‐tests unless otherwise specified. Survival curves were analyzed using the log‐rank (Mantel–Cox) test.

Sample sizes were not predetermined by statistical methods but were consistent with those used in previous studies (Grandison et al. 2009; Wei et al. 2024). Data distribution was assumed to be normal but was not formally tested. In *Drosophila* phenotype experiments, investigators were not blinded to group allocation during experiments or outcome assessment due to the nature of the experimental setup and practical limitations, consistent with standard practices in the field. In mass spectrometry experiments, data collection and analysis were performed in a blinded manner, and steps were taken to minimize potential batch effects.

## Author Contributions

F.W. conceived and designed the study, performed experiments, analyzed data, and drafted the manuscript. S.L. developed the metabolic flux calculation method. F.W. and Y.S. optimized the chemically defined diet. Y.S. and J.L. assisted in metabolomics data analysis. H.H. performed part of the experiments, analyzed the data, and contributed to manuscript revision. B.F. and F.W. contributed to manuscript revision. F.W. supervised the project. All authors reviewed and approved the final manuscript.

## Funding

This work was supported by the National Institutes of Health (R01CA193256), Duke Department of Pharmacology and Cancer Biology and Chinese Academy of Agricultural Sciences.

## Conflicts of Interest

The authors declare no conflicts of interest.

## Supporting information


**Supporting Information: 1** Full formulation of the chemically defined diet (CDD).

**Supporting Information: 2** Fructose labeling rates for the isotope‐labeled diet (U‐^13^C_6_‐Sucrose).

## Data Availability

All data supporting the findings of this study are available at https://github.com/LocasaleLab/MR_Tau_Ex_2025. Source data are provided in this paper. Metabolite data analysis was performed using MetaboAnalyst 6.0 (http://www.metaboanalyst.ca/ MetaboAnalyst/) and GENE‐E (https://software.broadinstitute.org/GENE‐E/) with pathway annotations from the Kyoto Encyclopedia of Genes and Genomes (KEGG) (http://www.genome.jp/kegg/). All scripts were implemented in Python 3.8. Source code is available at https://github.com/cmplab‐cimr/202508_Fangchao_MR_Tau_Exercise.
