# Supplementary material for: Gut Metabolism Links Precision Nutrition, Exercise, and Healthspan in Drosophila melanogaster
Source: Aging Cell. 2026 Jul 20;25(7):e70628. doi: 10.1111/acel.70628 (PMC13382535; doi:10.1111/acel.70628)
Supplement: Supplementary file 1 — Supporting Information: 1 Full formulation of the chemically defined diet (CDD). [file ACEL-25-e70628-s001.docx]

Chemically Defined Diet (CDD) formula:

|  | **Category** | **Ingredient** | **Amount per liter** | **Amount per 70mL** |
| --- | --- | --- | --- | --- |
| **Step 1** | Gelling Agent | Agar | 10 g | 0.7 g |
|  | Sugar | Sucrose | 25 g | 1.75 g |
|  | Metal Ions | CaCl_2_ * 6H_2_O | 1 mL | 70 µL |
|  |  | CuSO_2_ * 5H_2_O | 1 mL | 70 µL |
|  |  | FeSO_2_ * 7H_2_O | 1 mL | 70 µL |
|  |  | MgSO_4_ (anhydrous) | 1 mL | 70 µL |
|  |  | MnCl_2_ * 4H_2_O | 1 mL | 70 µL |
|  |  | ZnSO_4_ * 7H_2_O | 1 mL | 70 µL |
|  | Cholesterol | Cholesterol | 15 mL | 1050 µL |
|  | Amino Acids | Tyrosine | 0.93 g | 0.0651 g |
|  | Amino Acids | Histidine | 50 mL | 3.5 mL |
|  |  | Isoleucine | 50 mL | 3.5 mL |
|  |  | Methionine | 50 mL | 3.5 mL |
|  |  | Phenylalanine | 50 mL | 3.5 mL |
|  |  | Threonine | 50 mL | 3.5 mL |
|  |  | Valine | 50 mL | 3.5 mL |
|  | Water | Water (milli Q) |  | 11.06 mL |
| **Autoclave 15 minutes** | | | | |
| **Step 2** | Base | Buffer | 100 mL | 7 mL |
|  | Amino Acids | Arginine | 10 mL | 700 µL |
|  |  | Cysteine | 10 mL | 700 µL |
|  |  | Glutamate | 10 mL | 700 µL |
|  |  | Glycine | 10 mL | 700 µL |
|  |  | Lysine | 10 mL | 700 µL |
|  |  | Proline | 10 mL | 700 µL |
|  |  | Serine | 10 mL | 700 µL |
|  | Amino Acids | Alanine | 50 mL | 3.5 mL |
|  |  | Asparagine | 50 mL | 3.5 mL |
|  |  | Aspartate | 50 mL | 3.5 mL |
|  |  | Glutamine | 50 mL | 3.5 mL |
|  |  | Leucine | 50 mL | 3.5 mL |
|  |  | Tryptophan | 50 mL | 3.5 mL |
|  | Vitamin Solution |  | 21 mL | 1.47 mL |
|  | Folic Acid |  | 1 mL | 70 µL |
|  | Other Nutrients |  | 8 mL | 560 µL |
|  | Preservatives | Propionic Acid | 6 mL | 420 µL |
|  |  | Methyl 4-hydroxybenzoate | 15 mL | 1050 µL |

CDD prepare directions:

Prepare step 2 mixture and set aside;

Prepare step 1 mixture, adding everything but agar;

Add agar to step 1 mixture, stir using stir bar;

Autoclave step 1 mixture for 15 minutes;

Remove step 1 mixture from the autoclave, then combine with step 2 mixture and stir;

Quickly pipette diet into *Drosophila* vials (~5 mL diet/vial);

Allow diet to solidify/cool for roughly an hour, then cover vials and store food at 4 °C.

CDD Stock Solutions:

| **20 × Amino Acid Stocks:** | **Catalog No.** | **1 g/L** | **20 × Stock g/50 mL** | **Suspend in: (H_2_O unless**  **otherwise specified)** |
| --- | --- | --- | --- | --- |
| L-Phenylalanine | Sigma, P5482 | 1.01 | 1.01 |  |
| L-Isoleucine | Amresco, E803 | 1.12 | 1.12 |  |
| L-Leucine | Sigma, L8912 | 2.03 | 2.03 | 0.2 N HCl |
| L-Methionine | Amresco, E801 | 0.60 | 0.60 |  |
| L-Threonine | Sigma, T8441 | 1.11 | 1.11 |  |
| L-Valine | Amresco, 1B1102 | 1.20 | 1.20 |  |
| L-Alanine | Sigma, A7469 | 1.10 | 1.10 |  |
| L-Aspartic Acid | Alfa Aesar, A13520 | 1.17 | 1.17 | 0.5 N NaOH |
| L-Asparagine | Amresco, 94341 | 1.03 | 1.03 |  |
| L-Glutamine | Amresco, 0374 | 1.12 | 1.12 |  |
| L-Histidine | Amresco, 1B1164 | 0.65 | 0.65 |  |
| L-Tryptophan | Amresco, E800 | 0.32 | 0.32 |  |
| **100 × Amino Acid Stocks:** | **Catalog No.** | **1 g/L** | **100 × Stock g/50 mL** | **Suspend in: (H_2_O unless otherwise specified)** |
| L-Lysine HCl | Amresco, 0437 | 1.37 | 6.83 |  |
| L-Arginine HCl | Amresco, 0877 | 1.63 | 8.16 |  |
| L-Cysteine | Sigma, 30089 | 0.34 | 1.71 | 1 N HCl |
| L-Glutamic Acid NaH_2_0 | Alfa Aesar, A12919 | 1.52 | 7.59 |  |
| L-Glycine | Alfa Aesar, A13816 | 0.77 | 3.84 |  |
| L-Proline | Sigma, P5607 | 0.98 | 4.89 |  |
| L-Serine | Sigma, S4311 | 1.38 | 6.89 |  |
| **100 × Amino Acid Stocks:** | **Catalog No.** | **1 g/L** | **100 × Stock g/50 mL** | **Suspend in: (H_2_O unless otherwise specified)** |
| L-Lysine HCl | Amresco, 0437 | 1.37 | 6.83 |  |
| L-Arginine HCl | Amresco, 0877 | 1.63 | 8.16 |  |
| L-Cysteine | Sigma, 30089 | 0.34 | 1.71 | 1 N HCl |
| L-Glutamic Acid NaH_2_0 | Alfa Aesar, A12919 | 1.52 | 7.59 |  |
| L-Glycine | Alfa Aesar, A13816 | 0.77 | 3.84 |  |
| L-Proline | Sigma, P5607 | 0.98 | 4.89 |  |
| L-Serine | Sigma, S4311 | 1.38 | 6.89 |  |
| L-Tyrosine | Sigma, T8566 | Just add Tyrosine powder | |  |
| **Folic Acid Solution** | **Catalog No.** | **1 g/L** | **Stock g/50 mL** | **Suspend in** |
| Folic Acid | Sigma, F8758 | 0.0005 | 0.025 | 0.004 N NaOH |
| **Methyl 4-hydroxybenzoate Solution** | **Catalog No.** | **1 g/L** | **Stock g/50 mL** | **Suspend in** |
| Methyl 4-hydroxybenzoate | Sigma, H3647 | 1.5 | 5 | 95% EtOH |
| **Metal Ion Stocks** | **Catalog No.** | **1000 ×:** | **Stock g/50 mL** | **Suspend all in H_2_O** |
| CaCl_2_ * 6H_2_O | Sigma, 21108 | 250 g/L | 12.5 |  |
| CuSO_4_ * 5H_2_O | Sigma, C7631 | 2.5 g/L | 0.125 |  |
| FeSO_4_ * 7H_2_O | Sigma, F7002 | 25 g/L | 1.25 | Store at -20 °C |
| MgSO_4_ (anhydrous) | Sigma, M7506 | 250 g/L | 12.5 |  |
| MnCl_2_ * 4H_2_O | Sigma, M3634 | 1 g/L | 0.05 |  |
| ZnSO_4_ * 7H_2_O | Sigma, Z0251 | 25 g/L | 1.25 |  |
| **Cholesterol Solution Stocks** | **Catalog No.** | **1 × g/L** | **Stock g/50 mL** | **Suspend in EtOH** |
| Cholesterol | Sigma, C8253 | 0.3 | 1 g |  |
| **Vitamin Solution** | **Catalog No.** | **1 × g/L** | **Stock g/50 mL** | **Vitamin Solution** |
| Thiamine (aneurin) | Sigma, T4625 | 0.1000 | 0.005 | Thiamine (aneurin) |
| Riboflavin | Sigma, R4500 | 0.0500 | 0.003 | Riboflavin |
| Nicotinic acid | Sigma, N4126 | 0.6000 | 0.030 | Nicotinic acid |
| Ca pantothenate | Sigma, 21210 | 0.7750 | 0.039 | Ca pantothenate |
| Pyridoxine (HCl) | Sigma, P9755 | 0.1250 | 0.006 | Pyridoxine (HCl) |
| Biotin | Sigma, B4501 | 0.0100 | 0.001 | Biotin |
| **Vitamin Solution** | **Catalog No.** | **1 × g/L** | **Stock g/50 mL** | **Vitamin Solution** |
| Thiamine (aneurin) | Sigma, T4625 | 0.1000 | 0.005 | Thiamine (aneurin) |
| Riboflavin | Sigma, R4500 | 0.0500 | 0.003 | Riboflavin |
| Nicotinic acid | Sigma, N4126 | 0.6000 | 0.030 | Nicotinic acid |
| Ca pantothenate | Sigma, 21210 | 0.7750 | 0.039 | Ca pantothenate |
|  | | | | Combine all in H_2_O |
| **Other Nutrients Solution** | **Catalog No.** | **1 × g/L** | **Stock g/50 mL** | **Other Nutrients Solution** |
| Choline chloride | MP, 194639 | 0.0500 | 0.3125 | Choline chloride |
| Myo-Inositol | Sigma, I7508 | 0.0050 | 0.0315 | Myo-Inositol |
| Inosine | Sigma, I4125 | 0.0650 | 0.4065 | Inosine |
| Uridine | Sigma, U3003 | 0.0600 | 0.3750 | Uridine |
|  | | | | Combine all in H_2_O |
| **Buffer** | **Catalog No.** | **10 ×** | **Stock g/50 mL** | **Buffer** |
| Glacial Acetic Acid | Millipore, AX0074 | 30 ml/L | 1.5 mL | Glacial Acetic Acid |
| KH_2_PO_4_ | JT Baker, 3246 | 30 g/L | 1.5 g | KH_2_PO_4_ |
| NaHCO_3_ | Sigma, S8875 | 30 g/L | 0.5 g | NaHCO_3_ |
|  | | | | Combine KH_2_PO_4_ & NaHCO_3_ with  48.5 ml H_2_O, then add acetic acid |

Other reagents: Sucrose, Sigma, S7903; Agar, Caisson, A037; Propionic acid, Sigma, P5561; Inosine, Sigma, I4125; Uridine, Sigma, U3003; Adenosine, Sigma, A4036; Guanosine, Sigma, G6264; Cytidine, Sigma, C4654; Thymidine, Sigma, T1895. U-^13^C5-Methionine, Cambridge, CLM-893-H-0.1; U-^13^C6-Sucrose (Fructose), Cambridge, CLM-9811-PK.
